# Effective Interventions and Decline of Antituberculosis Drug Resistance in Eastern Taiwan, 2004–2008

**DOI:** 10.1371/journal.pone.0031531

**Published:** 2012-02-23

**Authors:** Yi-Ting Chen, Jen-Jyh Lee, Chen-Yuan Chiang, Gee-Gow Yang, Yeong-Chu Tsai, Yeong-Sheng Lee, Chih-Bin Lin

**Affiliations:** 1 Department of Internal Medicine, Buddhist Tzu Chi General Hospital, Hualien, Taiwan; 2 Department of Internal Medicine, School of Medicine, Tzu Chi University, Hualien, Taiwan; 3 International Union Against Tuberculosis and Lung Disease, Paris, France; 4 Division of Pulmonary Medicine, Department of Internal Medicine, Wan Fang Hospital, Taiwan; 5 Department of Internal Medicine, School of Medicine, Taipei Medical University, Taipei, Taiwan; 6 Department of Laboratory Medicine, Buddhist Tzu Chi General Hospital, Hualien, Taiwan; 7 Fourth Branch Office, Centers for Disease Control, Tainan, Taiwan; McGill University, Canada

## Abstract

**Background:**

The Taiwan health authority recently launched several tuberculosis (TB) control interventions, which may have an impact on the epidemic of drug-resistant TB. We conducted a population-based antituberculosis drug resistance surveillance program in Eastern Taiwan to measure the proportions of notified TB patients with anti-TB drug resistance and the trend from 2004 to 2008.

**Methods and Findings:**

All culture-positive TB patients were enrolled. Drug susceptibility testing results of the first isolate of each TB patient in each treatment course were analyzed. In total, 2688 patients were included, of which 2176 (81.0%) were new TB cases and 512 (19.0%) were previously treated cases. Among the 2176 new TB cases, 97 (4.5%) were retreated after the first episode of TB treatment within the study period. The proportion of new patients with any resistance, isoniazid resistance but not multidrug-resistant TB (resistant to at least isoniazid and rifampin, MDR-TB), and MDR-TB was 16.4%, 7.5%, and 4.0%, respectively, and that among previously treated cases was 30.9%, 7.9%, and 17.6%, respectively. The combined proportion of any resistance decreased from 23.3% in 2004 to 14.3% in 2008, and that of MDR-TB from 11.5% to 2.4%.

**Conclusions:**

The proportion of TB patients with drug-resistant TB in Eastern Taiwan remains substantial. However, an effective TB control program has successfully driven the proportion of drug resistance among TB patients downward.

## Introduction

In 2007, the notification rate for tuberculosis (TB) was 63.2 per 100,000 population in Taiwan. Among all regions, Eastern Taiwan (consisting of two counties, Hualien and Taitong) had the highest notification rate and mortality from TB, which was105 and 7.9 per 100,000 population, respectively. World Health Organization (WHO) and the International Union Against Tuberculosis and Lung Disease (The Union) launched the Global Project on Anti-tuberculosis Drug Resistance Surveillance in 1994, based on three principles: surveillance should be based on a samples of TB patients representative of all cases in a geographical setting; drug resistance must be clearly distinguished according to the history of TB treatment; and optimal laboratory performance must be ensured through a quality-assurance program [1], [2]. The latest guideline [3] for surveillance of drug resistance in TB indicates that surveillance, according to the International Health Regulations adopted by the 58^th^ World Health Assembly, is the systematic ongoing collection, collation and analysis of data for public health purposes and the timely dissemination of public health information for assessment and public health response as necessary. A surveillance system based on routine drug susceptibility testing (DST) of all TB cases provides continuous information on patterns and trends in anti-TB drug resistance and provides an opportunity to monitoring outbreaks of drug-resistant TB.

No official surveillance for anti-TB drug resistance has been conducted in Eastern Taiwan before Taiwan Centers for Disease Control (CDC) signed a contract with the mycobacteriology laboratory of Buddhist Tzu Chi General Hospital, which is the only laboratory in Eastern Taiwan that performs DST for *Mycobacterium tuberculosis (M. tuberculosis)*. All isolates of *M. tuberculosis* in Eastern Taiwan must to be sent to the mycobacteriology laboratory of Buddhist Tzu Chi General Hospital for DST, which forms the basis of the surveillance program in Eastern Taiwan. We analyzed the DST results of all relevant isolates from 2004 to 2008 to determine the proportion and trend of anti-TB drug resistance in Eastern Taiwan.

## Methods

### Setting and Procedures

Eastern Taiwan has a land mass of 8,144 km^2^ with a population of about 580,000. Table 1 shows the demographic profiles of the population in Eastern Taiwan in 2004–2008. The number of inhabitants who were homeless or infected with human immunodeficiency virus was small. One medical center, seven regional hospitals, and 29 public health centers occur in this region. Under the “Regional Reference Laboratory of Mycobacteriology” program of Taiwan CDC, sputum and clinical specimens from suspected and confirmed tuberculosis cases identified at any health facility in Eastern Taiwan should be sent to the mycobacteriology laboratory of Buddhist Tzu Chi General Hospital for culture and identification. Identification was done for all culture-positive strains, and DST was routinely conducted for the first isolate of every patient.

**Table 1:** Demographic profiles of the population in Eastern Taiwan, 2004–2008.

	2004	2005	2006	2007	2008
Population, n =	589522	586241	581260	576962	573282
Sex, %					
male	52.7%	52.6%	52.5%	52.3%	52.2%
female	47.3%	47.4%	47.5%	47.7%	47.8%
Age group (years), %					
0–19	25.3%	24.8%	24.3%	23.9%	23.3%
20–59	59.0%	59.5%	59.7%	59.9%	60.2%
≧60	15.7%	15.7%	15.9%	16.2%	16.5%
Indigenous people, %	28.4%	28.7%	28.9%	29.2%	29.4%
Homeless people, n (per 100,000 population)	166 (28.2)	109 (18.6)	80 (13.8)	53 (9.2)	26 (4.5)
HIV^a^ infection, n (per 100,000 population)	27 (4.6)	52 (8.9)	44 (7.6)	32 (5.5)	19 (3.3)

a HIV, human immunodeficiency virus.

Sputum collection, processing, decontamination, culture, and identification were performed following WHO-recommended methods, as reported previously [4]. All pretreated specimens were routinely inoculated into three types of media to increase yield rates: a BACTEC MGIT 960 culture tube, a 7H11 agar plate, and a Lowenstein-Jensen slant. Positive cultures in the BACTEC MGIT 960 culture tube were sub-cultured using Middlebrook 7H11 agar medium. The DST was performed using the indirect proportion method. The critical concentrations were 0.2 µg/mL for isoniazid (INH), 1.0 µg/mL for rifampicin (RMP), 5.0 µg/mL for ethambutol (EMB), and 2.0 µg/mL for streptomycin (SM), which were consistent with the WHO recommendation. Laboratory practices were not changed during the study period. The laboratory participated in DST proficiency testing coordinated by Taiwan CDC in 2006 and 2008 using strains provided by the Institute of Tropical Medicine, Antwerp, Belgium and was certified to be a quality-assured mycobacteriology laboratory in both rounds.

### Study design

All *M. tuberculosis* isolates recovered from notified TB cases diagnosed at all health care facilities in Eastern Taiwan from January 2004 to December 2008 were included in this study. A new TB case was defined as a patient with a newly registered episode of TB who had never been treated with anti-TB drugs for as much as 1 month. A previously treated TB case was defined as a patient with a newly registered episode of TB who had been treated with anti-TB drugs for 1 month or more. Previously treated cases included treatment after failure, treatment after default, relapses, and other previously treated cases. Patient treatment history was ascertained by reviewing medical records and cross-checking with the national TB registry of Taiwan CDC. If a patient had more than one DST result within each treatment episode, the first DST result was included. If a new TB case, after the first episode of treatment, was retreated because of failure, default or relapse during the study period, the DST result of the first isolate of the re-treatment episode was included to calculate the proportion of drug resistance among previously treated case.

### Statistical analysis

All data were entered into a computer using Microsoft Excel. The combined proportion of drug resistance among all cases, the proportion of resistance among new cases, and the proportion of resistance among previously treated cases were calculated separately. Categorical data were analyzed with the chi-square test using SPSS 11.0 (SPSS, Inc., Chicago, IL, USA). The trends of proportion of drug resistance in 2004–2008 were evaluated with the Mantel-Haenszel trend test using SAS [5]. A *p*-value<0.05 was regarded as statistically significant.

### Ethics

This study was approved by the Institutional Review Board of Tzu Chi General Hospital, Hualien, Taiwan. Informed consent was waived, as the study involved routine laboratory results and medical records, and no patient interviews were conducted.

## Results

**Figure 1:**
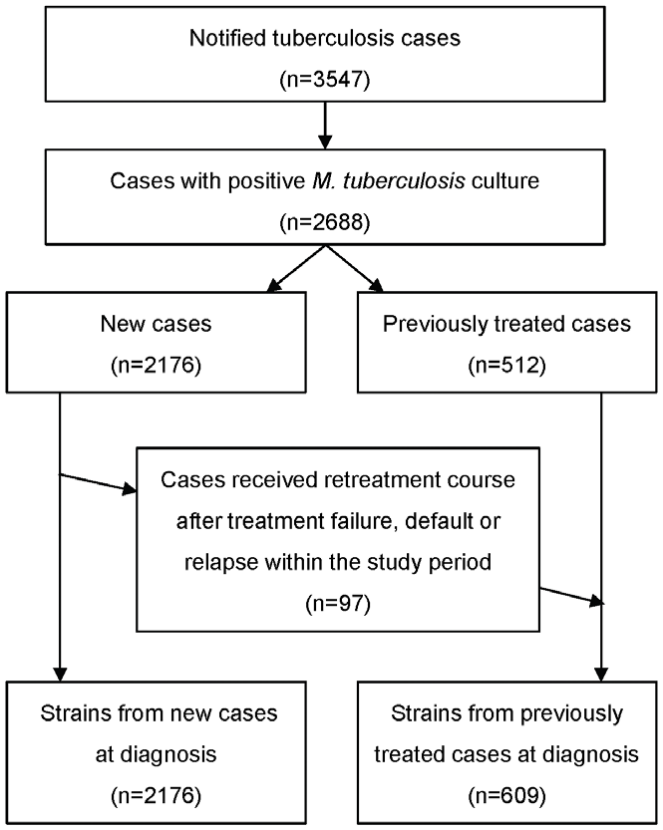
Flow diagram of tuberculosis subjects included in this study.

A total of 3547 tuberculosis patients were notified in 2004–2008 in Eastern Taiwan, in which 2688 were culture positive. The current study enrolled all of the 2688 patients of culture positive tuberculosis, accounted for 76% of all tuberculosis patients and 100% of culture positive cases in 2004–2008 in Eastern Taiwan. Among the 2688 cases, 2176 (81.0%) were new patients and 512 (19.0%) were previously treated patients. Among the 2176 new cases, 97 (4.5%) underwent re-treatment in 2004–2008 because of treatment failure, default, or relapse and were also included to calculate the proportion of anti-TB drug resistance among previously treated cases (Figure 1). A total of 2,785 non-duplicate isolates from 2688 TB patients were included in this study. Susceptibility of all isolates was determined.

Of the 2785 isolates, 2240 (80.4%) were susceptible to all first-line anti-TB drugs, 407 (14.6%) were resistant to INH, 216 (7.8%) were resistant to RMP, 48 (1.7%) were resistant to EMB, 261 (9.4%) were resistant to SM, and 195 (7.0%) were MDR-TB. Of the 2176 isolates from new cases, 1819 (83.6%) were susceptible to all fist-line drugs; 11.6% were resistant to INH, 4.5% were resistant to RMP, 1.1% were resistant to EMB,8.5% were resistant to SM, and 88 (4.0%) were MDR-TB. Of the 609 isolates from previously treated patients, 421 (69.1%) were susceptible to all first-line drugs; 25.5% were resistant to INH, 19.5% were resistant to RMP, 4.1% were resistant to EMB, 12.5% were resistant to SM, and 107 (17.6%) were MDR-TB. The proportion of resistance to any first-line drugs was significantly higher among previously treated cases than that among new cases (*p*<0.005) (Table 2).

**Table 2:** Prevalence of resistance to first line anti-tuberculosis drugs in Eastern Taiwan, 2004–2008, by type of case.

Drugs	All cases(n = 2785)	New cases(n = 2176)	Previously treated cases (n = 609)	*p*-value	Odds ratio(95% CI)
	Number (%)				
Isoniazid	407 (14.6)	252 (11.6)	155 (25.5)	<0.001	2.6 (2.1–3.3)
Rifampin	216 (7.8)	97 (4.5)	119 (19.5)	<0.001	5.2 (3.9–6.9)
Ethambutol	48 (1.7)	23 (1.1)	25 (4.1)	<0.001	4.0 (2.3–7.1)
Streptomycin	261 (9.4)	185 (8.5)	76 (12.5)	0.003	1.5 (1.2–2.0)
MDR^a^	195 (7.0)	88 (4.0)	107 (17.6)	<0.001	5.1 (3.8–6.8)

a Multidrug resistance, defined as resistance to at least isoniazid and rifampicin.

Table 3 shows the trend of combined resistance to the four first-line anti-TB drugs in all cases. The proportion of INH resistance, RMP resistance, EMB resistance, and MDR decreased significantly, but not SM resistance. The proportion of MDR-TB decreased steadily during the study period, from 11.5% in 2004 to 2.4% in 2008. Tables 4 and 5 show the proportion of resistance over the 5 years in new cases and previously treated cases, respectively. The trend in the decreased proportion of resistance was statistically significant for RMP resistance, EMB resistance, and MDR-TB among new cases and for resistance to all first-line drugs except SM among previously treated patients.

**Table 3:** Trends of combined resistance to first-line anti-tuberculosis drugs among all cases (n = 2785) in Eastern Taiwan, 2004–2008.

Drug^a^	2004 (n = 532)	2005 (n = 541)	2006 (n = 584)	2007 (n = 591)	2008 (n = 537)	*p*-value for trend
	Number (%)					
Any	124 (23.3)	108 (20.0)	116 (19.9)	120 (20.3)	77 (14.3)	0.002
INH	94 (17.7)	87 (16.1)	87 (14.9)	91 (15.4)	48 (8.9)	<.001
RIF	69 (13.0)	50 (9.2)	48 (8.2)	35 (5.9)	14 (2.6)	<.001
EMB	15 (2.8)	18 (3.3)	8 (1.4)	4 (0.7)	3 (0.6)	<.001
SM	63 (11.8)	42 (7.8)	54 (9.2)	56 (9.5)	46 (8.6)	0.232
MDR	61 (11.5)	45 (8.3)	42 (7.2)	34 (5.8)	13 (2.4)	<.001

a INH = isoniazid; RIF = rifampicin; EMB = ethambutol; SM = streptomycin; MDR = resistance to at least INH and RIF.

**Table 4:** Trend of resistance to first-line anti-tuberculosis drugs among new cases (n = 2176) in Eastern Taiwan, 2004–2008.

Drug^a^	2004 (n = 366)	2005 (n = 404)	2006 (n = 456)	2007 (n = 493)	2008 (n = 457)	*p*-value for trend
	Number (%)					
Any	65 (17.8)	62 (15.3)	75 (16.4)	89 (18.1)	66 (14.4)	0.420
INH	41 (11.2)	49 (12.1)	54 (11.8)	68 (13.8)	40 (8.8)	0.486
RMP	21 (5.7)	21 (5.2)	22 (4.8)	23 (4.7)	10 (2.2)	0.016
EMB	5 (1.4)	10 (2.5)	3 (0.7)	3 (0.6)	2 (0.4)	0.016
SM	40 (10.9)	26 (6.4)	39 (8.6)	41 (8.3)	39 (8.5)	0.577
MDR	18 (4.9)	19 (4.7)	19 (4.2)	23 (4.7)	9 (2.0)	0.049

a INH = isoniazid; RMP = rifampin; EMB = ethambutol; SM = streptomycin; MDR = resistance to at least INH and RMP.

**Table 5:** Trend of resistance to first-line anti-tuberculosis drugs among previously treated cases (n = 609) in Eastern Taiwan, 2004–2008.

Drug^a^	2004 (n = 166)	2005 (n = 137)	2006 (n = 128)	2007 (n = 98)	2008 (n = 80)	*p*-value for trend
	Number (%)					
Any	59 (35.5)	46 (33.6)	41 (32.0)	31 (31.6)	11 (13.8)	<0.001
INH	53 (31.9)	38 (27.7)	33 (25.8)	23 (23.5)	8 (10.0)	<0.001
RMP	48 (28.9)	29 (21.2)	26 (20.3)	12 (12.2)	4 (5.0)	<0.001
EMB	10 (6.0)	8 (5.8)	5 (3.9)	1 (1.0)	1 (1.3)	0.015
SM	23 (13.9)	16 (11.7)	15 (11.7)	15 (15.3)	7 (8.8)	0.552
MDR	43 (25.9)	26 (19.0)	23 (18.0)	11 (11.2)	4 (5.0)	<0.001

a INH = isoniazid; RMP = rifampin; EMB = ethambutol; SM = streptomycin; MDR = resistance to at least INH and RMP.

**Figure 2:**
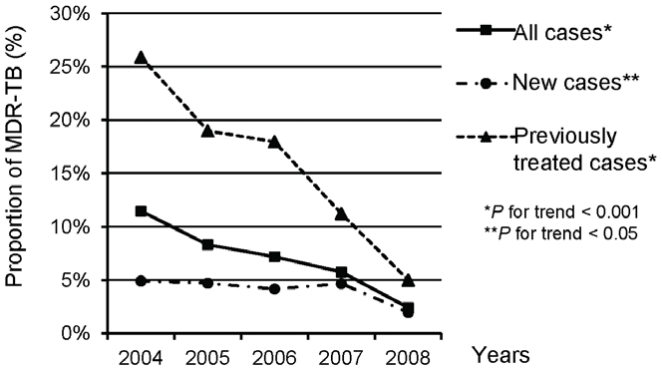
Trends of proportions of multidrug-resistant tuberculosis among new, previously treated and all tuberculosis cases, 2004–2008.

**Figure 3:**
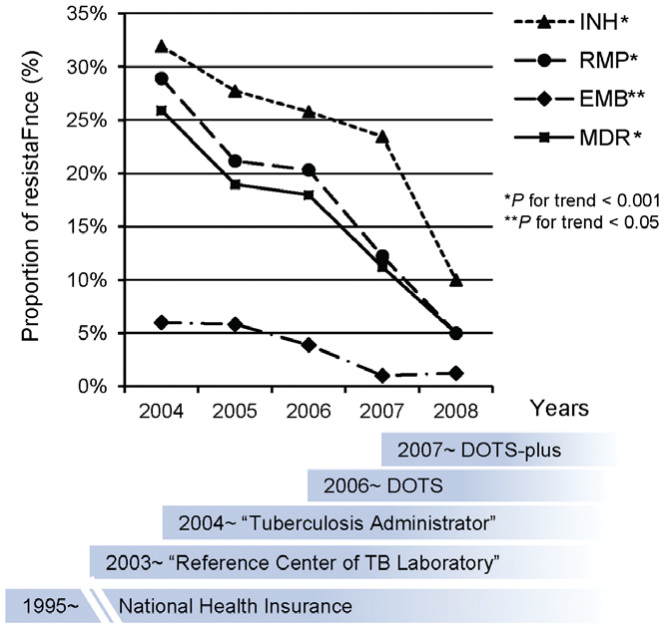
Decline of the drug resistance among previously treated tuberculosis patients and the calendar of introduction of new TB control interventions in Eastern Taiwan, 2004–2008.

The downward trend of resistance among previously treated patients was more prominent than that among new cases. Figure 2 shows that the proportion of MDR-TB among previously treated cases was 25.9% in 2004, which decreased consistently to 5.0% in 2008 with a cumulative reduction of 80.7%. The respective figures for that among new patients were 4.9%, 2.0% and 59.2%. Similar findings were observed for other first-line drugs except SM. Figure 3 shows the trends in the proportions of drug resistance among previously treated patients and the calendar for introducing new TB control interventions in Eastern Taiwan.

Of the 216 RMP-resistant isolates, 195 (90.3%) were MDR-TB, and the positive predictive value of RMP resistance for MDR-TB was 90.7% (88/97) among new cases and 89.9% (107/119) among previously treated cases.

## Discussion

Anti-TB drug resistance in Eastern Taiwan has been reported previously. The proportions of new and previously treated cases with resistance to any first-line anti-TB drugs in 2001–2002 were 16.8% and 64.5%, respectively, and those with MDR-TB were 2.1% and 45.2%, respectively [4].These data may not be representative of Eastern Taiwan, because isolates were mainly collected from patients treated at a medical center. Hospital-based studies on anti-TB drug resistance in Taiwan commonly reported a relative high proportion of drug resistance to any first-line drug, ranging from 30% to 52.4% [6], [7].

The present study was population-based, enrolled all culture-positive tuberculosis cases in 2004–2008 in Eastern Taiwan.The results revealed that the proportion of anti-TB drug resistance among notified TB cases in Eastern Taiwan remained substantial and that the proportion of new patients with MDR-TB was higher than the global average (4.0% vs. 2.9%). The recommended regimen for new TB patients in Taiwan was a 2-month intensive phase of INH, RMP, PZA, and EMB followed by a 4-month continuation phase of INH, RMP, and EMB [8]. SM was commonly used in the past, and the proportion of SM resistance was as high as 15.4% in 1971 [9]. SM has not been recommended for treating new TB patients for more than 20 years, and the proportion of SM resistance among all cases was 9.4% in this study. The proportions of resistance to INH, RMP, and EMB among previously treated cases were significant higher than those among new cases, with odds ratios of 2.6, 5.2, and 4.0, respectively (Table 2). The high proportions of resistance among previously treated patients highlights the importance of addressing the constraints of TB control program, particularly a patient's adherence to treatment.

The proportion of drug resistance in Eastern Taiwan decreased significantly in 2004–2008, particularly among previously treated patients. Similar findings were observed in recent studies conducted at medical centers in southern and central Taiwan [10], [11]; those studies were hospital-based and study population may not be representative, implying limitation of generalizability. Our study revealed 79.1% cumulative reduction in the proportion of MDR-TB among all cases in 5 years and 80.7% reduction among previously treated cases. Although the 4^th^ report of Global Project on Anti-tuberculosis Drug Resistance Surveillance demonstrated decrease in the proportion of MDR-TB among all cases from 1996 to 2005 in some settings [2], such as in Hong Kong (from 2.6% to 0.9%) and Singapore (from 0.8% to 0.3%), the magnitude of decline in anti-TB drug resistance was not as striking as our finding in Eastern Taiwan.

Trends in anti-tuberculosis drug resistance were regarded as an indicator of program performance [12], [13]. Several initiatives likely contributed positively to the decline in drug-resistant TB in Eastern Taiwan. First, the National Health Insurance (NHI) program was launched in 1995. The NHI program is government-run, compulsory, and inclusive, which improves access to care among people with low socioeconomic status. The proportion of the population insured under the NHI Program was 93% in 1996, and has been >97% since 2002 [14], [15]. Second, the “Regional Reference Laboratory of Mycobacteriology” program was initiated in 2003. DST has been systematically performed for notified tuberculosis cases, and the DST results provided guidance for treating tuberculosis. Third, the tuberculosis case manager (TCM) program was launched by Taiwan CDC in 2004. The TCM program provided financial support for health care facilities to recruit one or more TCM, depending on the number of TB patients served. The main responsibility of the TCM is tuberculosis case management and contacts examination. The proportion of TB patients successfully treated at Buddhist Tzu Chi General Hospital increased from 65.1% in 1997 to 88.4% after a full-time TCM was recruited in 2004 [16]. Fourth, the Department of Health of Taiwan endorsed “The Global Plan to Stop TB 2006–2015”, which aims to decrease the incidence of TB by 50% in Taiwan in 10 years. The Directly Observed Therapy (DOT) program was launched in April 2006. The proportion of smear positive cases managed with DOT was initially 65% in 2006, and increased to 92.6% in 2007 [17]. A preliminary analysis of data in 2006 revealed that treatment outcomes among patients with DOT were significantly better than those without DOT, namely patients treated with DOT had a higher proportion of sputum conversion in 3 months (47% vs. 33%, p<0.001), higher treatment success (75.1% vs. 51.6%, p<0.001), lower mortality rate (13.6% vs. 37.7%, p<0.001), and fewer defaults (3.5% vs. 5.0%, p<0.01) [17]. Further, the number of previously treated tuberculosis cases in present study has decreased from 166 in 2004 to 80 in 2008, likely due to effective case management rendering permanent cure of TB patients .. Fifth, Taiwan CDC launched a MDR-TB program (Taiwan MDR-TB consortium) in May 2007 to manage MDR-TB patients. A total of 225 MDR-TB patients have been enrolled since August 2008. The proportion of patients with a negative culture at month 18 was 87.6% among those who were enrolled, which was 1.6- (95% confidence interval, 1.4–2.0, *p*<0.0001) fold higher than those who were not enrolled [18].

The decline of drug resistance among new case was less striking than that among previously treated cases. Transmission of drug-resistant strains in the community prior to effective interventions may build up a sub-population infected with drug-resistant strains, who may develop drug-resistant tuberculosis over time. To clarify whether there is ongoing transmission of drug resistant tuberculosis in the community requires further researches.While our data revealed an encouraging decline in anti-tuberculosis drug resistance, continued surveillance on anti-tuberculosis drug resistance is essential to monitor sustainability of the downward trend.

The positive predictive value of RMP resistance for MDR-TB was 90.7% among new cases and 89.9% among previously treated cases, indicating that rifampin resistance could be used as a MDR-TB proxy, and that rapid testing of rifampin resistance may shorten the time for the diagnosis of MDR-TB.

In conclusion, the proportion of TB patients with drug-resistant TB in Eastern Taiwan remains substantial. However, an effective TB control program has successfully driven the proportion of drug resistance among TB patients downward, which is particularly prominent in previously treated cases. An effective TB control program must continue in Eastern Taiwan to further reduce anti-TB drug resistance.
